# Crystal Structure and Identification of Two Key Amino Acids Involved in AI-2 Production and Biofilm Formation in *Streptococcus suis* LuxS

**DOI:** 10.1371/journal.pone.0138826

**Published:** 2015-10-20

**Authors:** Yang Wang, Li Yi, Shaohui Wang, Hongjie Fan, Chan Ding, Xiang Mao, Chengping Lu

**Affiliations:** 1 College of Animal Science and Technology, Henan University of Science and Technology, Luoyang, China; 2 Shanghai Veterinary Research Institute, Chinese Academy of Agricultural Sciences, Shanghai, China; 3 Department of Life Science, Luoyang Normal University, Luoyang, China; 4 Key Lab of Animal Bacteriology, Ministry of Agriculture, Nanjing Agricultural University, Nanjing, China; Centre National de la Recherche Scientifique, Aix-Marseille Université, FRANCE

## Abstract

*Streptococcus suis* has emerged as an important zoonotic pathogen that causes meningitis, arthritis, septicemia and even sudden death in pigs and humans. Quorum sensing is the signaling network for cell-to-cell communication that bacterial cells can use to monitor their own population density through production and exchange of signal molecules. S-Ribosylhomocysteinase (LuxS) is the key enzyme involved in the activated methyl cycle. Autoinducer 2 (AI-2) is the adduct of borate and a ribose derivative and is produced from *S*-adenosylhomocysteine (SAH). AI-2 can mediate interspecies communication and in some species facilitate the bacterial behavior regulation such as biofilm formation and virulence in both Gram-positive and Gram-negative bacteria. Here, we reported the overexpression, purification and crystallographic structure of LuxS from *S*. *suis*. Our results showed the catalytically active LuxS exists as a homodimer in solution. Inductively coupled plasma-mass spectrometry (ICP-MS) revealed the presence of Zn^2+^ in LuxS. Although the core structure shares the similar topology with LuxS proteins from other bacterial species, structural analyses and comparative amino acid sequence alignments identified two key amino acid differences in *S*. *suis* LuxS, Phe80 and His87, which are located near the substrate binding site. The results of site-directed mutagenesis and enzymology studies confirmed that these two residues affect the catalytic activity of the enzyme. These *in vitro* results were corroborated *in vivo* by expression of the LuxS variants in a *S*. *suis* Δ*luxS* strain. The single and two amino acid of LuxS variant decreased AI-2 production and biofilm formation significantly compared to that of the parent strain. Our findings highlight the importance of key LuxS residues that influence the AI-2 production and biofilm formation in *S*.*suis*.

## Introduction

*Streptococcus suis* (SS) has emerged as an important zoonotic pathogen and is the causative agent of meningitis, arthritis, septicemia and even sudden death in pigs and humans [1]. Quorum sensing is used by bacterial cells for cell-to-cell communication, whereby they are able to monitor their own population density through production and exchange of signaling molecules [2]. In addition to having a metabolic role, some species have exploited LuxS/autoinducer-2 as a signaling molecule. The LuxS/autoinducer-2 signaling pathway forms an interspecies bacterial communication system for diverse bacterial species and is proposed to be involved in the regulation of many kinds of microbial behaviors [3]. The gene *luxS* is an integral component of the activated methyl cycle, with widespread conservation in prokaryotes [4]. LuxS is a vital component of the S-adenosyl-methionine metabolic pathway, where it catalyzes the production of 4,5-dihydroxy-2,3-pentanedione from S-ribosylhomocysteine (SRH). Recent studies have revealed that LuxS plays roles in the biosynthesis of AI-2, biofilm formation, cell metabolism and even resistance to the host immune response or antibiotics in several bacterial species [5, 6]. Since LuxS/AI-2 regulates the expression of bacterial virulence factors and biofilm formation, LuxS and the quorum sensing system are being investigated as novel antibiotic target [7].

Recently, a series of LuxS crystal structures have been reported from *Bacillus subtilis*, *Helicobacter pylori*, *Haemophilus influenzae* and *Deinococcus radiodurans*, with some LuxS structures both in their free forms and in complex with SRH [8–10]. These studies revealed that LuxS from diverse species share a similar overall fold with a highly conserved active site. LuxS exists as a homodimer and two identical active sites formed at the dimer interface. Two highly conserved residues, Cys-84 and Glu-57 (amino acid numbering in *E*. *coli* LuxS), have been demonstrated to act as critical residues for catalytic reaction. In addition, several other active-site residues including Ser-6, His-11 and Arg-39 are also important for LuxS activity as suggested in site-directed mutagenesis studies [11–13]. Plummer *et al* indicated that mutation of the critical amino acid G92D is responsible for the loss of AI-2 activity in *C*. *jejuni* [14].

In our previous study, we systematically investigated the functions of *S*. *suis* LuxS. AI-2 activity reaches the maximum in late exponential growth phase, and AI-2 production level is highly correlated with the transcription level of *pfs*, but not correlated with the level of transcription of *luxS*. Pfs is involved in methionine metabolism, regulating intracellular 5'-methylthioadenosine (MTA) and SAH levels. The addition of a low concentration of synthetic AI-2 was able to promote biofilm formation and host-cell adherence, while a *luxS* deletion mutant showed reduced biofilm formation, adherence and virulence [15, 16] [17, 18]. In the present study, we expressed, purified, and solved the crystal structure of *S*. *suis* LuxS. Our structural studies on LuxS allowed the identification of important residues (Phe80 and His87) for its catalytic activity, which is critical for AI-2 production and biofilm formation in *S*. *suis*.

## Materials and Methods

### Bacterial strains, culture conditions and plasmids

The strains, plasmids and culture conditions used in this study are describes in Table 1. The *luxS* mutant of HA9801 (Δ*luxS*) and the complementation strain (CΔ*luxS*) was constructed in a previous study [17]. The *S*. *suis* strains were grown at 37°C in Todd-Hewitt broth (THB) (Difco Laboratories, USA) and plated on THB agar containing 5% (vol/vol) sheep blood. THB medium supplemented with 1% fibrinogen was used in the biofilm assay. Antibiotics were used as follow: 100 μg/ml of spectinomycin (Spc) (Sigma) or 4 μg/ml of chloromycetin (Cm) (Sigma) were used for SS transformants, and 50 μg/ml of kanamycin (Kan) (Sigma) was applied to screen the *E*. *coli* transformants.

**Table 1:** Characteristics of bacterial strains, plasmids and primers used in this study

Strain, plasmid and primer	Relevant characteristics^a^ or sequence (5’—3’)	Source of references
Strains		
HA9801	Virulent strain of SS2 isolated from dead pig	Collected in our laboratory
Δ*luxS*	Mutation in *luxS* gene of HA9801; Cm	[17]
CΔ*luxS*	Complemented strain of Δ*luxS*; Spc; Cm	[17]
CΔ*luxS-*F80M	Complemented strain with plasmid encoded site directed revertant of F80M; Spc; Cm	In this study
CΔ*luxS-*H87Y	Complemented strain with plasmid encoded site directed revertant of H87Y; Spc; Cm	In this study
CΔ*luxS-*C82A	Complemented strain with plasmid encoded site directed revertant of C82A; Spc; Cm	In this study
*E*. *coli* BL21	DE3	Invitrogen
*V*.*harveyi* BB170	BB120 luxN::Tn5 (sensor 1^−^, sensor 2^+^) *V*. *harveyi*	[17]
*V*.*harveyi* BB120	Wild type *V*. *harveyi*	[17]
Plasmid		
pET28a+ vector	Prokaryotic protein expression vector; Kan	Novagen
pET-luxS	pET28a+ backbone with the luxS gene	In this study
pET-luxS-F80M	pET28a+ backbone with F80M site directed revertant LuxS	In this study
pET-luxS-H87Y	pET28a+ backbone with H87Y site directed revertant LuxS	In this study
pET-luxS-C82A	pET28a+ backbone with C82A site directed revertant LuxS	In this study
pSET2 vector	*E*. *coli-S*. *suis* shuttle vector; Spc	[17]
pSET-luxS	pSET2 backbone with the luxS gene	[17]
pSET-luxS-F80M	pSET2 backbone with F80M site directed revertant LuxS	In this study
pSET-luxS-H87Y	pSET2 backbone with H87Y site directed revertant LuxS	In this study
pSET-luxS-C82A	pSET2 backbone with C82A site directed revertant LuxS	In this study

The prokaryotic expression vector pET28a(+) (Novagen) encoding an N-terminal hexahistidine (His) was used to construct pET28-luxS. The *luxS* gene coding for the S-ribosylhomocysteinase (LuxS) was amplified from the genomic DNA extract of *S*. *suis* strain HA9801 by PCR and the primers used were 5’-CGCGGATCCATGAAAAAAGAAGTCACT-3’ and 5’-CCGGAATTCTTAGATTGGTTTTCTTTC-3’. It was digested with *EcoR* I and *BamH* I and ligated into *EcoR* I/*BamH* I-digested vector. The fidelity of the DNA construct with an N-terminal His_6_ tag was verified by DNA sequencing.

### Expression and purification of S.suis LuxS

*E*. *coli* BL21 (DE3) was transformed with pET28-luxS and grown in LB medium supplemented with 50 μg/mL kanamycin at 37°C until an optical density at 600 nm of 0.8 was reached. Expression of LuxS was induced by the addition of 0.1 mM isopropyl-β-D-thiogalactopyranoside (IPTG) for 5 h in the culture at 37°C. The cells were harvested by centrifugation and lysed by sonication (on ice) in 20 mM Tris-HCl (pH 8.0), 0.2M NaCl, 10 mM imidazole, 0.1% Triton X-100, and 1 mM phenylmethylsulfonyl fluoride. The crude lysate was clarified by two centrifugations at 60,000 g for 30 min at 4°C and filtration through a 0.45- mm membrane. Soluble protein was purified by HisTrap column (GE) followed by size-exclusion chromatography on a Superdex 200 FPLC column (Amersham Biosciences). Fractions containing LuxS were pooled and concentrated in an Amicon Ultra-15 filter (Millipore) using a 10-kDa cutoff membrane. Protein purity was established by sodium dodecyl sulfate-polyacrylamide gel electrophoresis (SDS-PAGE), and protein concentrations were determined using a bicinchoninic protein assay according to the manufacturer’s instructions (Pierce).

### Site-directed mutagenesis and complementation of luxS deficient variant

A QuikChange site-directed mutagenesis kit (Stratagene) was used to construct the LuxS mutant proteins according to the manufacturer’s instructions. The primer pairs used for the mutagenesis are listed in Table 2. A double mutant (F80M/H87Y) was prepared with the same method and primer using F80M mutant plasmid as the template. All mutations were verified by nucleotide sequences of the entire coding regions. Each recombinant protein was produced and purified as described above for parental LuxS. The same methods were used for site-directed mutagenesis of the complementation plasmid pSET2-C. The plasmids were electrotransformed into the Δ*luxS* mutant, electrotransformation and identification were performed according to our previously described method [17].

**Table 2:** Oligonucleotides used for the site-directed mutagenesis in this study.

primer	Relevant sequence (5’—3’)
H(87)-Y up	tgtcggacaggtttctatatgattatgtgggga
H(87)-Y down	tccccacataatcatatagaaacctgtccgaca
F(80)-M up	atcgactgctctcctatggggtgtcggacaggt
F(80)-M down	acctgtccgacaccccataggagagcagtcgat
C(82)-A up	tgctc tcctt tcggg gca cggac aggtt tccac
C(82)-A down	gtgga aacct gtccg tgc cccga aagga gagca

### Crystallization, data collection, structure determination, and refinement

The initial crystallization trials were set up with the commercially available Hampton Kits using the hanging-drop vapor diffusion method. Purified LuxS with a final concentrated to 10 mg/mL was mixed with an equal of reservoir buffer containing 0.2 M potassium-sodium tartrate, 0.1 M sodium citrate (pH 6.5), and 1 M ammonium sulfate and incubated at 18°C in hanging-drop vapor diffusion plates. Crystals of the native enzyme, with excellent diffraction, appeared within 3 to 7 days.

For data collection, a single crystal was first immersed into a cryoprotectant consisting of 17% glycerol and 83% reservoir solution for 3min. The crystal was then mounted on a nylon loop and flash-cooled in a nitrogen gas stream at 100 K. Full dataset was collected at 2.20 Å resolution using synchrotron radiations at the beamline BL17U of SSRF, Shanghai, China. The data for the protein crystal were processed using the programs MOSFLM [19] and SCALA [20]. The structure of LuxS was solved by molecular replacement using the program MolRep [21] with the *B*. *subtilis* LuxS as a search model (PDB code 2FQO). The structure was rebuilt and refined using COOT [22] and the REFMAC5 program [23]. Final models were validated using PROCHECK [24]. Structure figures were generated by PyMOL [25]. The final statistics for data collection and structural refinement are summarized in Table 3.

**Table 3:** Crysal structure parameters, data collection and refinement statistics.

Data collection	Parameter
Space group	P212121
Unit cell parameters (Å)	a = 74.89,b = 86.28,c = 103.49
Resolution range (Å)	38.2–1.93
Number of total reflections	683278
Number of unique reflections	50447
Completeness (%)	99.8
*R*_*merge*_ ^a^	0.069 (0.321)
Mean *I/σ* ^b^	6.5 (3.1)
R_free_ ^c^	0.24
R_work_ ^d^	0.20
RMS bond (Å)	0.015
RMS angle(°)	1.6

The values in parentheses are those for the highest resolution shell.

^*a*^ R_merge_ = Σ|*I*_*h*_−<*I*>_*h*_|/ Σ_h_, where I_h_ is the intensity of an individual measurement of the reflection and < *I* >_*h*_ is the mean intensity of the reflection.

^*b*^ *I/σ* is the mean of the intensity/sigma of the unique, averaged reflections.

^*c*^ R_work_ = Σ|*F*_*o*_−*F*_*c*_|/ Σ*F*_*o*_, for the 95% of the reflection data used in the refinement. *F*_*o*_ and *F*_*c*_ are the observed and calculated structure factor amplitudes, respectively.

^*d*^R_free_ is the equivalent of R_work_, except that it was calculated for a randomly chosen 5% test set excluded from the refinement.

### Metal ion analysis

Purified LuxS protein was concentrated to 10 mg/mL, and equal volumes of protein solution and flowthrough as the control were treated with 1 M HNO_3_. Metal ion analysis by inductively coupled plasma-mass spectrometry (ICP-MS) was carried out at the Nanjing Institute of Geography Limnology, Chinese Academy of Sciences.

### In vitro production of AI-2 and estimation of AI-2 concentration

*In vitro* AI-2 synthesis reactions were carried out at 37°C according to the method previously described [15, 17]. Briefly, AI-2 was produced by incubation with 1 mM S-adenosylhomocysteine (Sigma, USA) and 1 mg/ml of purified Pfs and LuxS for 1 h at 37°C in 10 mM sodium phosphate buffer at pH 8.0. Pfs protein was purified in our previous study and the protocol described in our previous report [17]. The AI-2 concentration was estimated using Ellman’s assay to quantify homocysteine concentration and measuring the absorbance at 412 nm.

### Kinetic characterization of mutant and native LuxS

The activities of mutant and wildtype LuxS were assayed according to a published procedure [26]. Briefly, LuxS activity was determined spectrophotometrically at 37°C by monitoring the formation of homocysteine using Ellman’s assay. The assay mixture contained increasing concentrations of S-adenosylhomocysteine (Sigma, USA), 10 mM sodium phosphate buffer (pH 7.5), 1 mg/mL of purified Pfs and LuxS. The initial velocity versus substrate concentration was fitted into the Michaelis-Menten equation using the enzyme kinetics module from SigmaPlot software to obtain the *k*_cat_ and *K*_M_ values.

### AI-2 bioassay

Autoinducer-2 (AI-2) level in cell-free culture media was measured using the reporter strain *V*. *harveyi* BB170 as described previously [15]. Briefly, Cell-free culture fluid (CF) was prepared by centrifugation at 12,000 × *g* for 10 min, and the resulting supernatant was further filtered through a 0.22-μm filter (Millipore, USA). A 100μl aliquot of each sample was added to white, flat-bottomed, 96-well plates (Thermo, USA). A positive control was obtained from overnight culture of BB120, and sterile medium was used as the negative control. AI-2 activity is expressed as the difference in relative light units compared with the level of luminescence induced by the positive control (*V*. *harveyi* BB120). The AI-2 activity of *V*. *harveyi* BB120 was set at 100%. Luminescence values were measured with a Tecan GENios Plus microplate reader in luminescence mode (TECAN, Austria). For a single experiment, the bioassay was performed at least in duplicate for each sample. Experiments were repeated at least three times.

### Biofilm plate assay

*S*. *suis* were tested for production of biofilm using the protocol described in our previous report [16, 17]. Briefly, an overnight culture of *S*. *suis* was diluted to OD600 of 0.2 into fresh THB medium and incubated at 37°C for 24 h without agitation before being stained with crystal violet. Medium alone served as a negative control. After fixing with methanol, staining was measured at 595 nm.

### Protein structure accession number

The final coordinates and its related structure factors were deposited into the Protein Data Bank with the accession number of 4XCH.

## Results

### Identification of divalent cations bound to native LuxS by ICP-MS

LuxS is a homodimeric iron-dependent metalloenzyme containing two identical tetrahedral metal-binding sites. To determine the species of metal ion located in the *S*. *Suis* LuxS active site, ICP-MS experiment was performed. The result showed that the protein sample (15 mg/mL) contained 1.31 μg/mL Cu^2+^, 0.24 μg/mL Mn^2+^,1.16 μg/mL Ni^2+^ and 26.1 μg/mL Zn^2+^. Based on the concentration of the protein and its molecular weight, the calculated molar ratio of Zn^2+^ ion to each LuxS monomer is about 1:1, indicating that each LuxS monomer possesses one Zn^2+^. The ICP-MS data was consistent with the result of Ruzheinikov [10], but different from the result of Rajan *et al* [13]. The discrepancy perhaps has relation with the bacterial growth conditions.

### Structural characterization of LuxS

**Fig 1:**
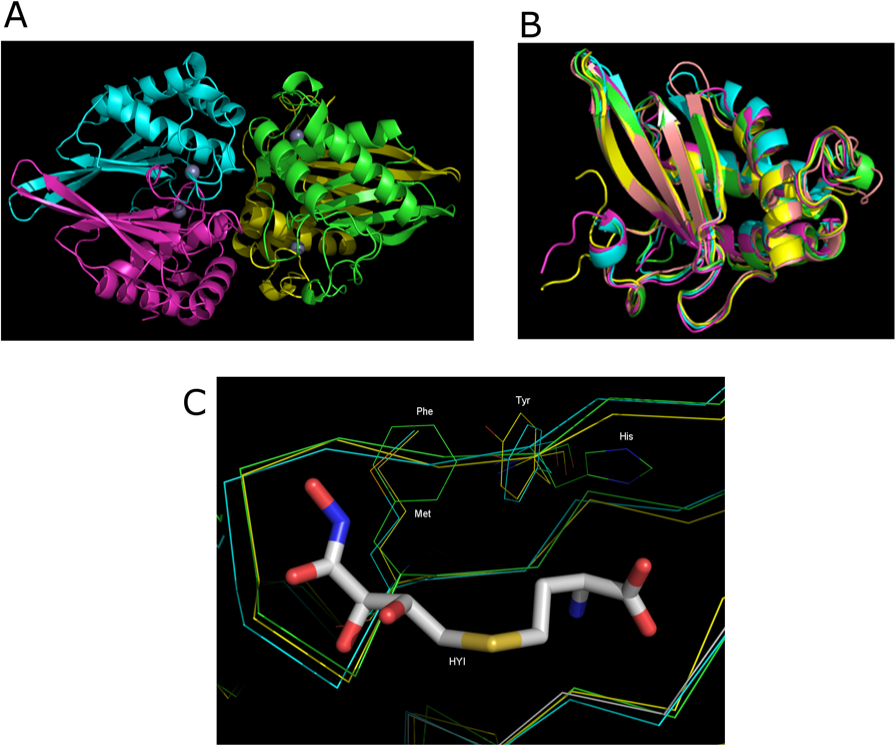
X-ray crystallographic structure of *S*. *suis* LuxS. (A) The overall structure of the LuxS. Four LuxS monomers are in one asymmetry unit, the different color coding represents different monomers. Zn is represented as a large sphere. (B) The structural comparisons of *S*. *suis* LuxS with four known structures of LuxS. The four known structures of LuxS proteins are sampled from Protein Data Bank. The different color coding as the LuxS structure of *Heamophilus influenzae* (1JOE, yellow),*Deinococcus radiodurans* (1VH2, green), *Helicobacter subtilis* (1J6X, cyan), *Bacillus subtilis* (2FQO, grey). (C) Residue phe80 and his87 were shown, the Met and Tyr were from 1JOE and 1J6X. HYI was compound (2S)-2-amino-4-[(2R,3R)-2,3-dihydroxy-3-N-hydroxycarbamoyl-propylmercapto]butyric acid from structure 2FQO. Four structures were shown, including 1VH2 (green), 1JOE (yellow), 1J6X (cyan) and 2FQO (grey).

Four LuxS monomers were present in one asymmetry unit (Fig 1A). From the result of size exclusion chromatography experiment, LuxS exists as a dimer in solution (data not shown), which is consistent with other reports. The tetramer observed in the crystal structure may be due to crystal packing. Each monomer was found to be composed of a four anti-parallel beta sheets and four anti-parallel alpha helices, which is similar to known LuxS crystal structures from other bacterial species including *Bacillus subtilis* (PDB code 2FQO), *Deinococcus radiodurans *(PDB code 1VH2), *Haemophilus influenzae* (PDB code 1JOE) and *Helicobacter pylori* (PDB code 1J6X). These are arranged in the order H1-S1-S2-H2-S3-S4-H3-H4. A ribbon shows highlighting the overall structural conservation with RMSD between LuxS from *S*.*suis* and LuxS from other species is shown in Fig 1B, where no significant conformational changes were found between the structures from these four different species.

### Bioinformatic analyses of LuxS proteins from other species

**Fig 2:**
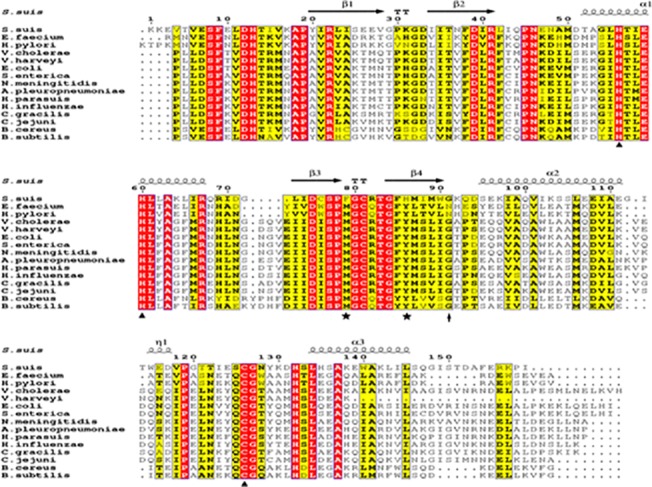
Structure-based multiple sequence alignment of LuxS homologues. Multiple alignments of LuxS homologue from *S*. *suis* HA9801 with the related LuxS proteins. The multiple alignment was conducted using ClustalW2 (http://www.ebi.ac.uk/Tools/clustalw2/index.html), and the figure was generated with the program ESPript 2.2 (http://espript.ibcp.fr/ESPript/cgi-bin/ESP ript.cgi). Sequence alignment was performed for the various LuxS sequences, which included *S*.*suis*, *E*.*facecium*, *H*.*pylori*, *V*.*cholerae*, *V*.*harveyi*, *E*.*coli*, *S*.*enterica*, *N*.*meningitidis*, *A*.*pleuropneumoniae*, *H*.*parasuis*, *H*.*influenzae*, *C*.*gracilis*, *C*.*jejuni*, *B*.*cereus*, *B*.*subtilis*. The secondary structure of the SS LuxS protein is shown in top. α: α-helix; β: β-sheet; T: β-turns/coils. Three conserved zinc-binding sites (His57, His61, and Cys127) are indicated with black triangles. A critical amino acid G81 was also very conserved (highlighted with an arrow), which was recently found to be one of the important residues in the active site for AI-2 production in *Campylobacter jejuni* (Plummer et al., 2011). The Cys82 is the catalytic active residue of the LuxS protein. The distinct residues are indicated by labeling with black stars.

Amino acid sequence alignment revealed that *S*. *suis* LuxS shares 22.5 to 68% sequence identity with LuxS proteins from several other bacterial species [27], including *Enterococcus facecium*, *H*. *pylori*, *Vibrio cholerae*, *Vibrio harveyi*, *Escherischia coli*, *Salmonella enterica*, *Neisseria meningitidis*, *Actinobacillus pleuropneumoniae*, *Haemophilus parasuis*, *H*. *influenzae*, *Campylobacter gracilis*, *Campylobacter jejuni*, *Bacillus cereus and B*. *subtilis*. Three highly conserved residues critical for zinc binding (His57, His61, and Cys127) were clearly observed in these LuxS proteins (Fig 2). It was recently reported that Gly92 of LuxS from *C*. *jejuni* is required for AI-2 production, and this conserved amino acid was also observed in other species except for in *Listeria*, *Chromohalo*, *Staphylococcus* and *Helicobacteria* LuxS proteins [14].

Sequence alignment of 14 LuxS proteins from a wide range of bacterial species revealed that the residues surrounding the substrate at the active site are strictly conserved (Fig 2). Our LuxS structure showed that two residues in active site were different from LuxS proteins from other species, i.e., a methionine to phenylalanine mutation at position 80 and a tyrosine to histidine mutation at position 87 (Fig 1C).

### Kinetic studies on LuxS mutants

To clarify the potential roles of Phe80 and His87 in the substrate binding and catalysis, these two residues were mutated Met and Tyr (F80M and H87Y). The proteins were expressed and purified, and their catalytic kinetic parameters were analyzed (Table 4). The results showed that the F80M mutant had approximately 7-fold decreased activity compared to wild type, with *k*_cat_ also decreasing approximately 5-fold, while K_M_ was essentially unchanged. The activity of the H87Y mutant decreased by approximately 37-fold, with a 4.3- and 9.4-fold decrease in K_M_ and *k*_cat_, respectively. The double mutant (F80M/H87Y) resulted in 42-fold lower activity and with a 3.7- and 11.3-fold decrease in K_M_ and *k*_cat_, respectively. The catalytic residue Cys82 was also mutated to Ala and, as expected, the C82A mutant lost all catalytic activity.

**Table 4:** Kinetic parameters for SAH, wild-type and mutant enzymes, measured at pH7.2

Protein	K_M_ (μM)	*K*_cat_(s^-1^)	*K*_cat_ */K*_M(_M^-1^/ s^-1)^
Wild-type	16.4±0.5	0.102±0.01	6.21×10^3^
F80M	22.3±0.5	0.020±0.003	8.82×10^2^
H87Y	70.8±4	0.011±0.002	1.53×10^2^
F80M/H87Y	60.4±4	0.009±0.002	1.49×10^2^
C82S	57.7±2	0.010±0.002	1.68×10^2^
C82A	inactive		

### Complementation of ΔluxS with luxS variants

**Fig 3:**
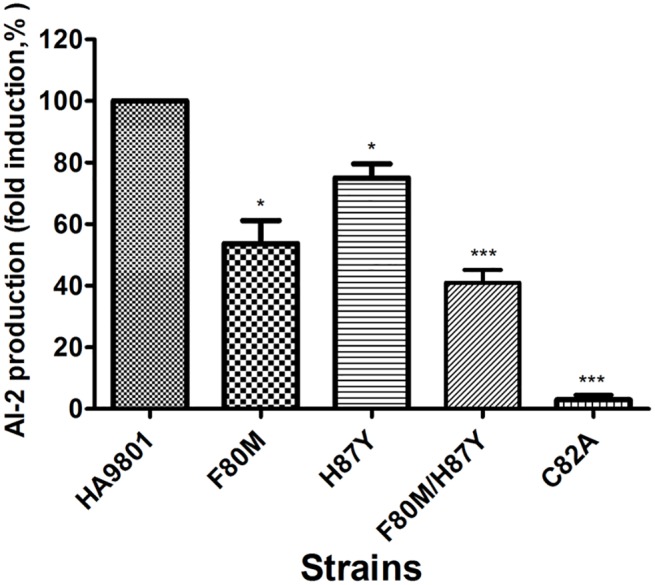
Measurement of AI-2 activity of the different complementation of Δ*luxS* with *luxS* variants. *S*.*suis* Δ*luxS* cells were transformed with pSET2 carrying *luxS* variants. AI-2 activity of cell-free culture fluids in the stationary exponential phase was measured using the *V*. *harveyi* bioluminescence assay. AI-2 activity determination is expressed as relative light units and compared with the level of luminescence produced by the positive control (*V*. *harveyi* BB120). AI-2 activity of *S*. *suis* HA9801 was set to 100% activity for normalization. Sterile THB medium served as a negative control. Values are means from three independent experiments. Error bars indicate standard deviations (p < 0.05).

To determine the functional importance of the F80M, H87Y and double (F80M/H87Y) mutants in AI-2 production, the Phe-80 and His-97 was mutated to a methionine and tyrosine in the complementation plasmid pSET-*luxS*. Complementation of Δ*luxS* with *luxS* variants that were introduced of the plasmid carrying the revertant *luxS* gene decreased production of AI-2 compared with the parent strain (Fig 3). As expected, the C82A mutant was defective in AI-2 production, at a similar level to the medium-only control (THB), while AI-2 production in the F80M, H87Y and the double mutants were decreased by 54%, 75% and 41%, respectively, compared with the parental strain, suggesting the F80 and H87 mutations affected the production of AI-2.

### Evaluation of biofilm formation in S. suis ΔluxS and luxS complemented variants

**Fig 4:**
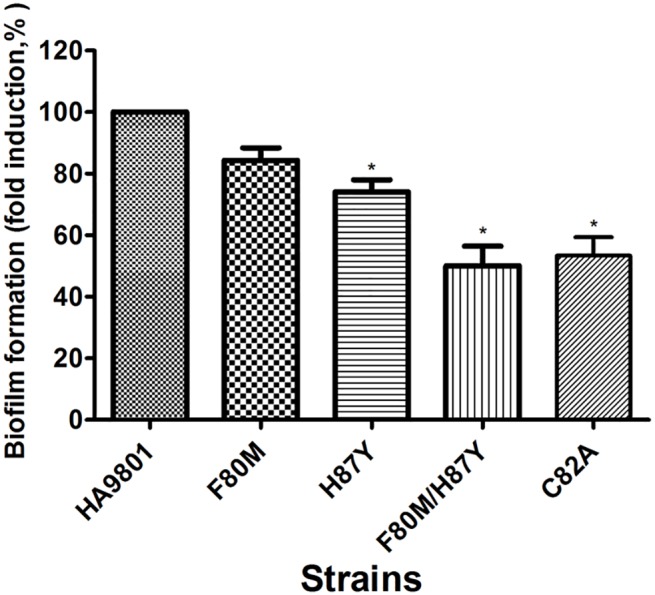
Quantitative determination of biofilm formation of the different complementation of Δ*luxS* with *luxS* variants. All strains were cultured in THB medium supplemented with 1% fibrinogen. Each strain was tested in 8 wells of a 96-well microtiter plate. HA9801, F80M, H80Y, C80A and F80M/H80Y refer to the WT strain and the complementation of Δ*luxS* with F80M, H80Y, C80A and F80M/H80Y point or double mutantion *luxS* variants. Negative control (NC) wells contained broth only. The columns represent the means and standard deviations of four or more experiments. The asterisk showed significant difference (p < 0.05).

To investigate differences in biofilm formation between parental and mutant strains, biofilm formation was quantitatively analyzed using a 96 microtiter plate assay (Fig 4). Biofilm formation was measured in THB medium supplemented with 1% fibrinogen. The C82A, F80M, H87Y and double mutant (F80M/H87Y) had slightly decreased biofilm formation at 0.53-fold, 0.85-fold, 0.73-fold and 0.50-fold (*P* < 0.05), respectively, when compared to the parental strain HA9801.

## Discussion

Much progress has been made over the last decade in understanding the role of LuxS, which is a metabolic enzyme with a key role in the production of the AI-2 molecule used for signaling in some species. The LuxS protein from *S*. *suis* is evolutionarily conserved and a *luxS* deletion mutant strain showed reduced biofilm formation, adherence and virulence; phenotypes that have been fully characterized in two previous studies [17, 18]. The LuxS crystal structure reported here provides further information on its functional role in the life cycle of *S*. *suis*.

The *S*. *suis* LuxS protein reported here is very similar to previously reported LuxS structures [8]. Sequence alignment of known LuxS structures from other bacterial species indicated that *S*. *suis* LuxS has two important residues that differ from other species, which are residues Phe80 and His87 located at the substrate binding site. Our enzymatic studies showed that these two residues play important roles in the kinetics of the enzymatic reaction and in the stabilization of the enzymatic intermediates. Based on our LuxS structure, Phe80 and His87 are responsible for substrate binding. His87 forms hydrogen bonds with Asp76B, which further interacts with the substrate through hydrogen bonding with a carboxyl group (Fig 1C). An optimal interaction between substrate and protein may be impaired when His87 was mutated to other residues, resulting in low binding affinity of the substrate and a decrease in the catalytic efficiency of the enzyme. Phe80 is close to the sugar ring of the substrate, which might form hydrophobic interactions between the substrate, in order to position the substrate optimally (Fig 1C). Site directed mutagenesis has been used in previous studies to explore the active site residues and significant reductions in enzyme activity have been associated with mutagenesis of these amino acids [11, 12].

The results of the AI-2 bioassay and biofilm formation demonstrated that these two residues in LuxS affect physiological functions of *S*. *suis* (Figs 3 and 4). Residual enzyme activity was observed in these mutants, which explained the detection of AI-2 following *in vitro* incubation of recombinant mutant enzyme with an excess of SRH. As suggested by the enzyme kinetic parameters of the Phe80 and His87 mutants, impaired production of AI-2 of the mutants was also observed in the complemented strains, leading to impaired ability to form biofilm. The *luxS* gene has been reported to play important roles in both metabolism and quorum sensing in a number of bacteria [4],however *luxS* mutation investigated here are clearly associated with altered biofilm formation, AI-2 production [17] and the role of AI-2 mediated quorum sensing in *S*. *suis* remains uncertain [28]. Plummer *et al* indicated that the mutation of the critical amino acid G92 to D is responsible for the loss of AI-2 activity in *C*. *jejuni* [14]. Furthermore, in our previous study, the Δ*luxS* mutant did not produce AI-2, and biofilm formation was significantly decreased in a microtiter plate assay [17, 18]. In agreement with previously published findings on the *luxS* mutants, the present study found that mutation of Phe80 and His87 resulted in decreased AI-2 and biofilm formation [14], revealing that Phe80 or His87 substitution in *S*. *suis* LuxS was responsible for a significant reduction in enzymatic activity and partial loss of AI-2 production and the ability to form biofilms. Here we have identified an enzyme (SS LuxS protein) with intermediate activity, which is neither fully functional nor non-functional, which may help in identifying other roles played by AI-2 quorum sensing in a wide range of bacterial species. Given that the amino acid responsible for this partly loss of AI-2 and biofilm, we speculate that these amino acids mutation allow for the maintenance of relatively normal spatial structure and are response to normal enzyme function. Collectively, our studies provide further information for the design of new antimicrobial drugs for the important amino acid against *S*. *suis* that target LuxS.

In this study, we reported the crystal structure of LuxS from *S*. *suis*, which was found to be similar to other reported *luxS* crystal structures. The ICP-MS data provided support for Zn^2+^ as a component of the active site of LuxS in *S*. *suis*. Sequence alignment of LuxS from other bacterial species showed that S. suis *LuxS* differs by two residues in the substrate binding site compared with other LuxS proteins. Site-directed mutagenesis of these two residues resulted in a decrease in K_M_ and *k*_cat_. To explore whether these mutants affected AI-2 production and biofilm formation, two complemented strains carrying each mutant were constructed. The results suggested that the decrease in AI-2 production and biofilm formation are caused by these F80M and H87Y mutations.
